# Overflow metabolism at the thermodynamic limit of life: How carboxydotrophic acetogens mitigate carbon monoxide toxicity

**DOI:** 10.1111/1751-7915.14212

**Published:** 2023-01-11

**Authors:** Maximilienne T. Allaart, Martijn Diender, Diana Z. Sousa, Robbert Kleerebezem

**Affiliations:** ^1^ Department of Biotechnology Delft University of Technology Delft The Netherlands; ^2^ Laboratory of Microbiology Wageningen University & Research Wageningen The Netherlands

## Abstract

Carboxydotrophic metabolism is gaining interest due to its applications in gas fermentation technology, enabling the conversion of carbon monoxide to fuels and commodities. Acetogenic carboxydotrophs play a central role in current gas fermentation processes. In contrast to other energy‐rich microbial substrates, CO is highly toxic, which makes it a challenging substrate to utilize. Instantaneous scavenging of CO upon entering the cell is required to mitigate its toxicity. Experiments conducted with *Clostridium autoethanogenum* at different biomass‐specific growth rates show that elevated ethanol production occurs at increasing growth rates. The increased allocation of electrons towards ethanol at higher growth rates strongly suggests that *C. autoethanogenum* employs a form of overflow metabolism to cope with high dissolved CO concentrations. We argue that this overflow branch enables acetogens to efficiently use CO at highly variable substrate influxes by increasing the conversion rate almost instantaneously when required to remove toxic substrate and promote growth. In this perspective, we will address the case study of *C. autoethanogenum* grown solely on CO and syngas mixtures to assess how it employs acetate reduction to ethanol as a form of overflow metabolism.

## Graphical abstract

Microorganisms have a pivotal role in the production of biofuels. Here, we explain how microorganisms that consume (waste) carbon monoxide can be pushed to produce bioethanol.

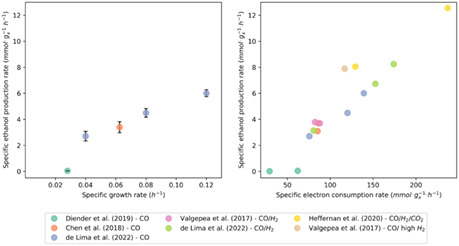

## INTRODUCTION

Carbon‐fixating acetogenic species have been characterized to convert CO_2_, H_2_ and CO to predominantly acetate and ethanol using the Wood–Ljungdahl pathway (WLP) (Wood, 1991; Wood et al., 1986). This conversion contributes substantially to the global carbon cycle as autotrophic acetogens fix CO_2_ into biologically available molecules. Additionally, these microorganisms provide us with a novel avenue for reducing greenhouse gas emissions and producing liquid fuels from gaseous waste streams such as syngas (Heffernan et al., 2020; Marcellin et al., 2016; Molitor et al., 2016). To steer industrial gas fermentation efficiently, mechanistic understanding of the factors driving cellular metabolism is required.

Among the different substrates for gas fermentation, carbon monoxide in particular proves to be a highly interesting substrate. It contains a substantial amount of energy in the form of low‐redox‐potential electrons (E^0^^′^ = −520 mV) that can be directly transferred to ferredoxin. Reduced ferredoxin can, among other things, be used for the generation of a cation gradient using the Rnf or Ech complex (Buckel & Thauer, 2018). These complexes allow acetogens to harvest energy from catabolic reactions that proceed close to thermodynamic equilibrium (Schuchmann & Müller, 2014). In catabolic pathways, electrons from the substrate are transferred to electron carriers such as NAD(P)(H), FAD(H) or ferredoxin and reallocated to different electron accepting reactions. This facilitates the evolutionary development of a wide variety of catabolic pathways and products. Generally, the catabolic flux can be carried by (I) a long, energy‐efficient pathway or by (II) a shorter pathway with a lower ATP yield that can run at a higher overall rate (Costa et al., 2006; Molenaar et al., 2009). Shifts in cellular strategy as a function of the (imposed) growth rate have been observed in cell types of different branches of the phylogenetic tree and are characterized by the utilization of energy‐inefficient pathways at high catabolic rates (Basan et al., 2015; Costa et al., 2006; Liberti & Locasale, 2016). This phenomenon is referred to as overflow metabolism. Different hypotheses have been posed to explain this effect, such as efficient proteome allocation, limited space in the cell membrane or cytosol, maximization of the ATP flux rather than yield, an upper limit to Gibbs energy dissipation or the number of active constraints on growth (Basan et al., 2015; de Groot et al., 2019; Flamholz et al., 2013; Goel et al., 2015; Molenaar et al., 2009; Niebel et al., 2019). Switching from balanced to overflow metabolism proceeds almost instantaneously, implying that the enzymes needed for both efficient and inefficient pathways must always be expressed (Bruggeman et al., 2020; Molenaar et al., 2009). The capacity to shift between balanced metabolism and overflow metabolism is a trait that provides flexibility and resilience towards ever changing environmental conditions.

In summary, overflow metabolism is the exploitation of a short, energetically inefficient metabolic route even when available nutrient concentrations are not limiting growth. This mechanism is used to optimally use cellular resources with the aim to maximize the biomass‐specific growth rate. Growth on gaseous substrates (H_2_/CO_2_, CO) is typically associated with highly variable substrate influxes and it has been recognized that ethanol production in gas‐fermenting organisms is controlled by redox and thermodynamics rather than gene or protein expression levels (Cotter et al., 2009; Mahamkali et al., 2020; Richter et al., 2016). To assimilate these findings, we pose that the reduction of acetate to ethanol is a form of overflow metabolism during acetogenic gas fermentation. This hypothesis will be assessed using a case study of *Clostridium autoethanogenum*, a model organism for carbon monoxide fermentation, using literature and thermodynamic analyses. We aim to solidify the theory by identifying overflow‐related traits in other carboxydotrophic acetogens.

## CARBON MONOXIDE DEHYDROGENASE IS THE FIRST STEP IN CO DETOXIFICATION

Acetogenic catabolism is highly dependent on carbon monoxide‐sensitive metalloenzymes such as NiFe‐ or FeFe‐hydrogenases to form intermediates for growth and to maintain cellular redox balance (Mock et al., 2015; Schuchmann et al., 2018; Wang et al., 2013). Carbon monoxide is known to bind metal clusters in enzymes and inhibit binding of their native substrates (Menon & Ragsdale, 1996). Therefore, carbon monoxide toxicity should be mitigated efficiently by carboxydotrophic organisms to prevent the ferredoxin pool to become completely reduced or key enzymes to get inhibited. Carbon monoxide is detoxified by the carbon monoxide dehydrogenase enzyme (CODH), which catalyses the oxidation of CO to CO_2_ upon reduction of ferredoxin at a high specific activity (Ragsdale, 2008; Svetlitchnyi et al., 2001). The maximum rate of diffusion of CO into the cell is defined by the concentration gradient between intra‐ and extracellular CO. Estimating the CO turnover rate using previously reported CODH activities and the absolute quantification of CODH concentration in *C. autoethanogenum* (Svetlitchnyi et al., 2001; Valgepea et al., 2022), we find that this number is in the same order of magnitude as the flux of CO diffusion into the cell in a CO‐saturated broth (Abrini et al., 1994) (for calculation, see Box 1). This suggests that CO is detoxified to CO_2_ immediately upon entering the cell, limiting inhibitory effects of CO on metalloenzymes. However, the capacity of the CODH to rapidly oxidize CO, even when diffusion into the cell is maximal, causes highly variable incoming fluxes of reduced ferredoxin. If the electrons carried by reduced ferredoxin cannot be re‐allocated equally fast, this could lead to a metabolic arrest. To understand the effects of fluctuating reduced ferredoxin formation rates and how they can be mitigated, the metabolism and physiology of carboxydotrophic acetogens have to be examined.

BOX 1 **Cellular CODH capacity and CO diffusion into the cell**

The orders of magnitude of the capacity of the CODH enzyme and the maximum rate of diffusion of CO into a cell give a clear perspective on the efficiency of the CODH enzyme for CO detoxification. These rates can, respectively, be calculated from a number of biological and physicochemical parameters.

**Calculating the activity of CODH**

In this calculation, kinetic parameters and molecular weight ($MW_{\text{CODH}}$) reported for CODH2 of *Carboxydothermus hydrogenoformans* are used (Svetlitchnyi et al., 2001). The concentration of CODH in *C. autoethanogenum* was quantified by Valgepea et al. (2022). The weight of a bacterial cell ($m_{\text{cell}}$) was assumed to be 1 picogram. The following parameters were used to calculate the CODH activity:

**Table:** 

Parameter	Value	Unit
Specific activity	$2.3\times 10^{-1}$	${\mathrm{mol}}_{\mathrm{CO}}\;s^{-1}\;g_{\text{CODHII}}^{-1}$
$c_{\text{CODH}}$	$122\times 10^{-9}$	$\mathrm{mol}\;g_{x}^{-1}$
$MW_{\text{CODH}}$	$129\times 10^{-3}$	$g_{x}\;{\mathrm{mol}}^{-1}$

$$\begin{array}{c} \text{CODH activity}=\text{Specific activity}\times c_{\text{CODH}}\times MW_{\text{CODH}}\\ \times m_{\text{cell}}=3.63\times 10^{-15}\;\mathrm{mol}\;s^{-1} \end{array}$$

**Calculating the rate of CO diffusion into the cell**

Here, the diffusion rate of CO into the cell was calculated at 25°C. It was assumed that the diffusion coefficient in the cell membrane is equal to the diffusion coefficient in water. Gas diffusion measurements in natural ester mixtures confirm that this assumption will give an estimate in the correct order of magnitude (Ye et al., 2019). The following parameters were used to calculate the CODH activity:

**Table:** 

Parameter	Value	Unit
$D_{\mathrm{CO}}$	$2.03\times 10^{-9}$	$m^{2}\;s^{-1}\;$
$c_{\mathrm{CO}}^{*}$	$9.85\times 10^{-4}$	$\mathrm{mol}\;L^{-1}$
$L_{\text{cell}}$	$5\times 10^{-9}$	m
$D_{\text{cell}}$	$5\times 10^{-7}$	m
$D_{\text{membrane}}$	$75\times 10^{-10}$	m

The diffusion flux can be calculated using Fick's law:

$$J=-D\frac{\mathrm{dc}}{\mathrm{dx}}$$

with $\mathrm{dc}=c_{\mathrm{CO}}^{*}$ (assuming the CO concentration in the cell is zero) and $\mathrm{dx}=D_{\text{membrane}}$. Then:

$$J=2.67\times 10^{-4}\;\mathrm{mol}\;m^{-2}\;s^{-1}$$

To convert this to the flux per cell, the surface area of the cell was calculated assuming the cell is a cylindrical rod. The diffusion flux was multiplied by the cell surface area, giving:

$$\mathrm{CO}\;\text{diffusion rate}=2.3\times 10^{-15}\;\mathrm{mol}\;s^{-1}$$

## HIGHER DILUTION RATES LEAD TO HIGHER ETHANOL PRODUCTION RATES IN *C. autoethanogenum*

**FIGURE 1:**
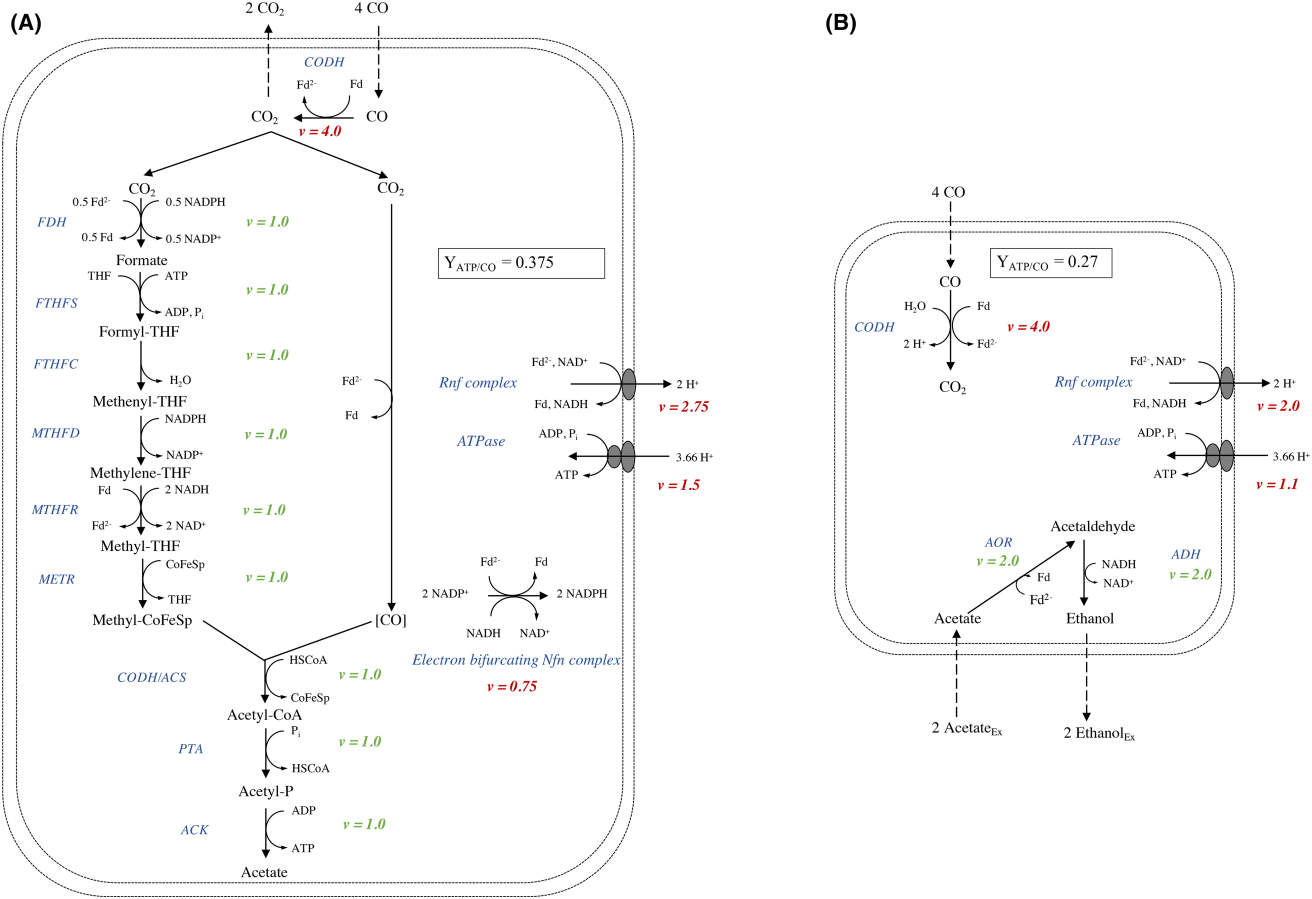
Energy‐efficient (A) versus energy‐inefficient (B) CO catabolism in *C. autoethanogenum* (Mock et al., 2015; Wang et al., 2013; Wood et al., 1986). Energy‐efficient CO fermentation leads to the production of acetate via the Wood–Ljungdahl or reductive acetyl‐CoA pathway. Inefficient catabolism comprises the uptake of external acetate and reduction to ethanol with CO as electron donor. In both catabolic pathways, the electron bifurcating Rnf complex is pivotal for harvesting ATP through buildup of a proton gradient. The catabolic stoichiometries and fluxes through the enzymatic reactions in both (A and B) are represented per 4 mol of CO.

*C. autoethanogenum* is an acetogen that employs the Wood–Ljungdahl pathway for carbon fixation. This pathway leads to the production of acetate and ethanol as main products. In light of the objective to convert waste gas into (bio)fuels, substantial efforts have been made to shift the product spectrum of *C. autoethanogenum* and closely related species towards ethanol production, for example, through medium optimization, changing the gas composition and metabolic engineering (Klask et al., 2020; Liew et al., 2017; Valgepea et al., 2018). The biochemistry of the Wood–Ljungdahl pathway allows for the highest ATP yield when acetate is produced as final product, due to the combination of ATP conservation from a cation gradient and substrate‐level phosphorylation. This makes diverting the acetyl‐CoA flux away from acetate towards ethanol via metabolic engineering approaches inherently difficult. Acetate formation on the one hand and the conversion of acetate to ethanol on the other can also be viewed as two separate metabolic strategies. Acetate formation from CO via the WLP is the longer, more efficient, strategy (Y_ATP/CO_ = 0.375 mol/mol_CO_) and the independent reduction of acetate to ethanol using CO is the shorter, inefficient strategy (Y_ATP/CO_ = 0.27 mol/mol_CO_) (Figure 1, Table 1). This perspective leads to the hypothesis that autotrophic acetogens, even though they operate their catabolism much closer to thermodynamic equilibrium than for example sugar fermenters or aerobic microorganisms, can exhibit overflow metabolism.

**TABLE 1:** Free energy change, ATP yield per mol of electron donor based on the enzymes depicted in Figure 1 and percentage of the free energy harvested in ATP.

Stoichiometry	ΔG^01^ (kJ/mol_Ed_)	Y_ATP/Ed_ (mol/mol_Ed_)	Y_ATP, max_ (mol/mol_Ed_)	ΔG_harvest_ (kJ/mol_Ed_)	# reactions (–)	ΔG_dis_ (kJ/reaction)
$2\;CO_{2}+4\;H_{2}\to C_{2}H_{4}O_{2}+2\;H_{2}O$	−13.8	0.240	0.31	78%	14	−0.9
$4\;\mathrm{CO}+2\;H_{2}O\to C_{2}H_{4}O_{2}+2\;{\mathrm{CO}}_{2}$	−33.8	0.375	0.75	50%	12	−5.6
$2\;\mathrm{CO}+C_{2}H_{4}O_{2}\to C_{2}H_{6}O+2\;{\mathrm{CO}}_{2}$	−41.0	0.273	0.91	30%	4	−14.4

*Note*: The ATP yield per electron donor was calculated assuming an Rnf complex stoichiometry of 1 H+ extruded per electron transferred and a H+/ATP stoichiometry of 3.66. To calculate the maximum ATP yield, it was assumed that 45 kJ of Gibbs free energy is required to produce one mole of ATP. The number of reactions was calculated by counting the number of carbon molecule‐altering biochemical steps and multiplying them by their respective fluxes.

**FIGURE 2:**
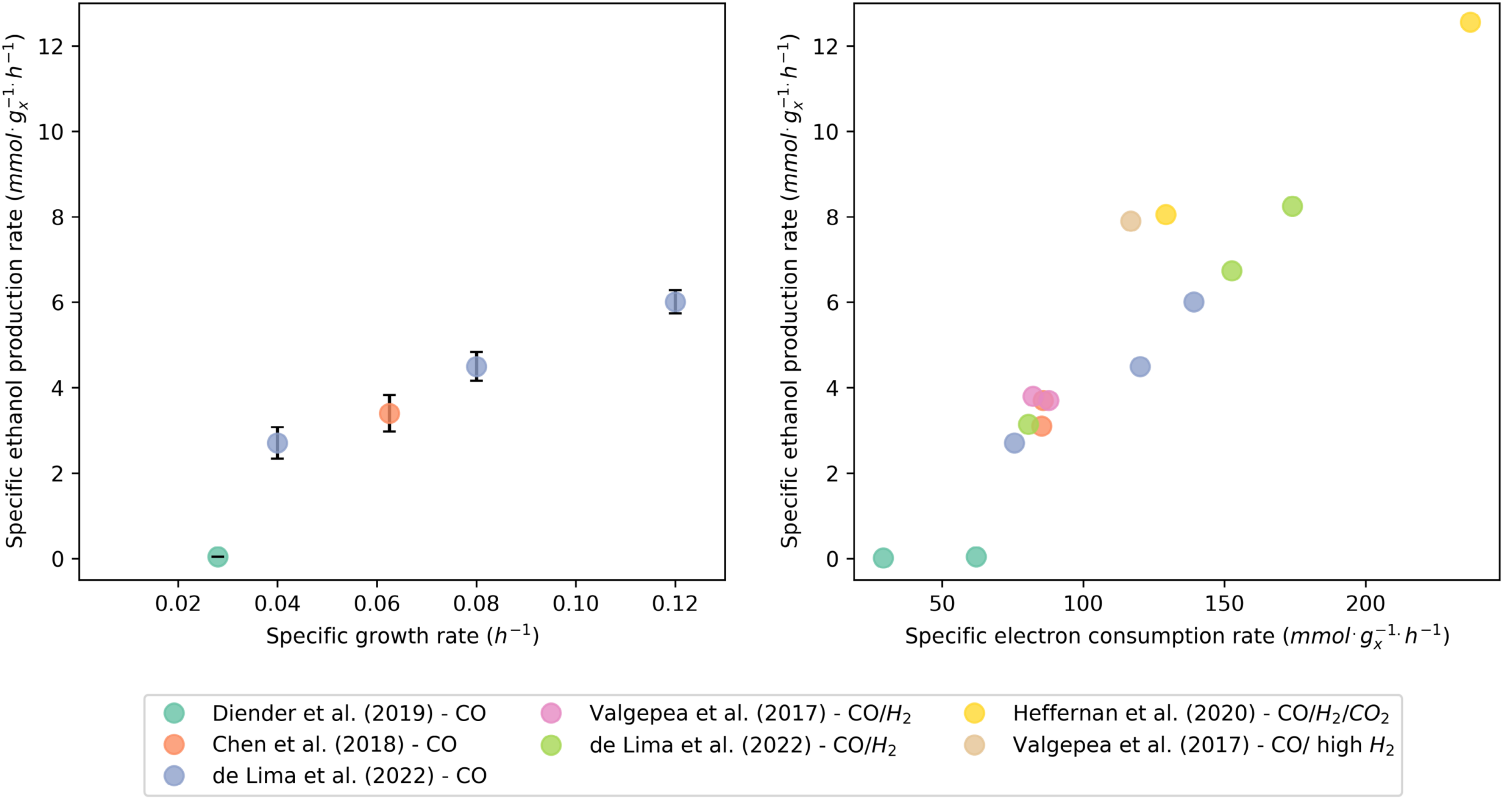
Dependency of the biomass‐specific ethanol production rate (*q*_EtOH_) on specific growth and electron uptake rate of *C.* *autoethanogenum*. Left panel: *q*_EtOH_ data were obtained from three independent studies (Chen et al., 2018; de Lima et al., 2022; Diender et al., 2019) and plotted as a function of the reported μ. All studies were performed in gas–liquid mass transfer‐limited continuous cultures fed with CO as only carbon and electron source, either in chemostat or in a bubble column. One study (Diender et al., 2019) was executed at higher pH than the other two studies (6.2 vs. 5). Low pH is known to trigger solventogenesis (Richter et al., 2016), which may explain the slight diversion of *q*_EtOH_ in this study as compared to the trend through the other data sets. Right panel: Chemostat studies fed with mixtures of CO, H_2_ and CO_2_ were compiled by plotting the electron uptake rate ($\sum \left(q_{\mathrm{CO}}+q_{H_{2}}\right)\times 2$) versus the observed ethanol production rate. Seven different data sets of continuous cultures with *C. autoethanogenum* were included in this analysis (Chen et al., 2018; de Lima et al., 2022; Diender et al., 2019; Heffernan et al., 2020; Valgepea et al., 2017, 2018).

Independent CO‐limited continuous cultivation studies show that at higher dilution rates and consequently higher growth rates (μ = D), more ethanol is produced (Figure 2A). The dilution rate linearly correlated with the biomass‐specific CO uptake rate, as increasing dilution rates were matched with an increased mass transfer rate by applying a faster stirring speed (de Lima et al., 2022). The authors note that ‘These observations are not trivial as faster growth demands more energy while reduced products like ethanol […] consume redox cofactors that acetogens could otherwise use for ATP generation’ (de Lima et al., 2022). Overflow metabolism provides a likely explanation for their observations. Beside this, transcriptome analysis reveals that the expression levels of the catabolic genes involved in production of both acetate and ethanol were expressed at similar levels at different growth rates. However, higher transcript levels for genes involved in the synthesis of biomass and for the Rnf complex (used both in efficient and inefficient pathways) were found at higher growth rates (de Lima et al., 2022). The transcriptome data are in agreement with results reported in earlier studies, where catabolic transcript levels were also independent of growth conditions (Mahamkali et al., 2020; Richter et al., 2016). This means that changes in product spectrum do not arise from changes in catabolic protein concentrations but are driven by condition‐imposed changes in metabolite levels.

Thus, the key characteristics of overflow metabolism are distinguished in continuous cultures of *C. autoethanogenum*:

1. The catabolic flux is increasingly diverted to an energy‐inefficient pathway at increasing dilution rates (Figure 2A)
2. No significant changes in transcriptome or proteome of the central carbon metabolism, including acetate conversion to ethanol, are observed at different growth rates, but genes involved in biosynthesis are upregulated.

To expand our understanding of acetogen overflow metabolism on gaseous substrates, it should be noted that higher growth rates generally correspond to higher electron uptake rates. Inclusion of steady‐state data obtained in chemostats fed with CO as well as H_2_ by summing the biomass‐specific CO and H_2_ rates to an overall electron uptake rate (*q*_electron_) also resulted in an apparent linear correlation between *q*_electron_ and *q*_EtOH_ (Figure 2B). This suggests that increased ethanol production generally allows for increased electron uptake rates. Like other types of overflow metabolism, acetate reduction to ethanol is thus exploited to sustain higher metabolic rates (Molenaar et al., 2009).

## THEORETICAL PATHWAY ANALYSIS — YIELD VERSUS RATE TRADE‐OFF

To understand the drivers of overflow metabolism, we can view the selection of the metabolic strategy as a yield versus rate trade‐off. The difference between energy‐efficient and energy‐inefficient metabolism is in fact the extent to which the free energy generated in a metabolic pathway can be harvested by the microorganism. Harvesting energy as ATP allows for re‐allocation to other cellular processes such as growth and maintenance. However, dissipating free energy is required to enable flux in the process. In other words, energy dissipation generates driving force towards the production of a certain metabolite. As stated above, ethanol is used as an electron sink when the influx of electrons increases (Figure 2B). As the production of ethanol requires the consumption of a reduced ferredoxin (Figure 1), less reduced ferredoxin will be available for the generation of a chemiosmotic gradient from which ATP can be harvested. Thus, on average, more energy is dissipated per reaction step in ethanol formation and cells producing ethanol operate further away from the thermodynamic limit (Table 1). Following the flux–force relationship, reactions that are further away from thermodynamic equilibrium generally proceed at a higher flux (Beard & Qian, 2007; Flamholz et al., 2013; Peng et al., 2020). In acetogenic metabolism, imposing a higher electron flux in turn leads the system to move further away from thermodynamic equilibrium by diverting more of the incoming electrons to ethanol rather than acetate (Figure 2). Furthermore, it has been observed that chemostat cultures of *C. autoethanogenum* grown on H_2_/CO_2_ mixtures, the metabolism with the lowest energy dissipation, washed out when doubling the dilution rate from 0.02 h^−1^ to 0.04 h^−1^ whereas CO‐fed cultures could be grown at up to 0.12 h^−1^ (de Lima et al., 2022; Heffernan et al., 2020). These observations all confirm that there is a relationship between energy dissipation and flux (Rodríguez et al., 2008). Thus, the product spectrum of acetogenic syngas fermentation is subject to a rate versus yield trade‐off, which in essence is a trade‐off between harvesting and dissipating Gibbs free energy. This complies with the conclusions in earlier work that stated that the metabolism of the acetogens *C. autoethanogenum* and *C. ljungdahlii* is not subject to transcriptional regulation (Mahamkali et al., 2020; Richter et al., 2016). The outcome of the trade‐off between dissipating and harvesting energy can therefore be controlled when imposing a certain amount of energy dissipation by imposing a certain flux. This can be achieved by controlling the biomass‐specific growth rate or the biomass‐specific electron uptake rate (Figure 2).

## EXPANDING THE HYPOTHESIS: CO OVERFLOW METABOLISM IN THE ACETOGEN ECOLOGICAL NICHE

Verification of the overflow hypothesis in other carboxydotrophic acetogens is limited by the lack of comparable cultivations described in literature. Few studies cultivate in chemostats fed with CO as only carbon and energy source, let alone at different growth rates. However, careful examination of various sources does point at the exhibition of overflow metabolism in other species that are genetically highly similar to *C. autoethanogenum* and harbour the same set of enzymes for acetate and ethanol production (Brown et al., 2014; Lee et al., 2019; Liou et al., 2005). First of all, ethanol has been proposed to be an overflow product of which the production is thermodynamically controlled in *C. ljungdahlii* (Richter et al., 2016). This control is, however, linked to a certain critical concentration of acetic acid and reduced electron carriers inside the cell. Similarly, a genome‐scale metabolic model of *C. ljungdahlii* predicts ethanol to be an overflow product (Liu et al., 2019). In both studies, the experimental data to link overflow metabolism to growth or electron uptake rate are lacking. In a batch bioreactor with *Clostridium* sp. AWRP decreasing the gas flow rate led to less ethanol and more acetate production, whereas increasing the stirring speed at the same gas flow rate led to acetate consumption and ethanol formation (Lee et al., 2019). By controlling the gas flow rate and stirring speed, both the mass transfer‐ and thereby the biomass‐specific electron uptake rates are controlled. This is a clear indication that this species exploits overflow metabolism to manage increasing electron influxes. Even in batch bottle cultivations of *Clostridium carboxidivorans* a negative correlation between acetate concentration and agitation speed can be distinguished, whereas alcohol (ethanol, butanol and hexanol) concentrations keep increasing with increasing agitation (Phillips et al., 2015).

More generally, organisms capable of growth on CO‐rich gases have an additional electron sink beside acetate. For *C. autoethanogenum* and *C. ljungdahlii*, this is ethanol formation (Liu et al., 2019; Richter et al., 2016), whereas *C. carboxidivorans* can produce ethanol, butanol and hexanol (Hurst & Lewis, 2010; Phillips et al., 2015). Furthermore, the well‐studied thermophilic CO fermenter *Moorella thermoacetica* is capable of proton reduction to H_2_ using electrons from CO, providing it with a non‐organic electron sink (Martin et al., 1983). Lastly, the gut bacterium *Eubacterium limosum* has been characterized to produce ethanol, butyrate and butanol from CO (Park et al., 2017). Interestingly, *Acetobacterium woodii* is only known to produce acetate and suffers from CO at increasing CO partial pressures (Bertsch & Müller, 2015). The catabolic routes that these microorganisms employ for the reallocation of increased electron fluxes require organic acids as a precursor. Acetate is the product of their efficient catabolism, but other weak acids (i.e. butyrate, hexanoate) can originate from the fermentation of organic carbon sources. This gives a perspective on the ecological niche of CO‐utilizing species, namely that organic acids are plentiful in their native environments. The abundance of CO‐consuming acetogens in soils and the fact that *C. autoethanogenum* particularly was isolated from a rabbit gut confirms this perspective (Abrini et al., 1994; Levy et al., 1981). Additionally, this suggests that higher concentrations of acetate boost the uptake capacity of CO consumers. Indeed, increased overflow product formation has been observed in different studies upon acetate supply (Diender et al., 2019; Kwon et al., 2022; Park et al., 2017; Richter et al., 2016; Wang et al., 2013; Xu et al., 2020). Besides an increased capacity for electron reallocation, overflow products can be re‐used when substrate is scarcer (Bertsch et al., 2016; Enjalbert et al., 2017; Liu et al., 2020).

To provide a full picture of overflow metabolism in *C. autoethanogenum* and other CO‐consuming acetogens, future experimental work should be directed at showing the effect of increasing CO supply at (very) low extracellular acetate concentrations. If the theory applies, the microorganisms will sustain lower biomass‐specific CO uptake rates than when acetate is available in abundance.

## OVERFLOW METABOLISM IS USED TO PREVENT OVER‐REDUCTION OF ELECTRON CARRIER POOLS

To prevent toxicity effects of CO, carboxydotrophic microorganisms need reliable mechanisms to deal with variable substrate influxes. The high turnover rates of the CODH enzyme ensure low intracellular CO concentrations. However, the conversion of CO to CO_2_ via the CODH also results in production of reduced ferredoxin. The rate of oxidation of CO therefore directly influences the redox state of the cell, as ferredoxin needs to be re‐oxidized at an equally high rate to maintain cellular homeostasis. The primary mechanism for ferredoxin reoxidation in carboxydotrophic acetogens is through the generation of a cation gradient via the Rnf complex or similar enzymes. This bifurcating enzyme transfers the electrons from reduced ferredoxin to NAD^+^, generating NADH that again must be re‐oxidized. NADH oxidation through acetate formation requires 12 enzymatic steps (Table 1), whereas only four steps are employed in the acetate reduction to ethanol. Considering a situation where the CO influx is high, ethanol formation has the additional advantage of consuming a reduced ferredoxin for the formation of acetaldehyde. Thus, ethanol formation provides a route that contributes both to ferredoxin and NADH reoxidation and that requires few enzymes compared to acetate formation. If the enzymes for ethanol production would not be available, all the enzymes of the WLP would either have to be quickly upregulated to sustain a higher flux or always be highly expressed to be able to deal with changing conditions. Expressing only two additional enzymes (AOR and ADH) to increase the capacity of re‐oxidizing electron carriers is more efficient from a resource allocation point of view. The continuous availability of the enzymes for acetate reduction to ethanol, despite non‐solventogenic conditions (Richter et al., 2016) fit the theory that this pathway is used for overflow metabolism. Consistent presence of the machinery of this pathway allows to directly deal with sudden increases in CO supply and prevent over‐reduction of the electron carrier pools. This is also reflected in the observation that start‐up of CO‐fed systems is easier when acetate or even other carboxylic acids are added to the culture medium (Perez et al., 2013; Xu et al., 2020). The overflow function of these enzymes is also supported by the observation that acetogens lacking the AOR enzyme, such as *A. woodii*, are prone to inhibition by CO as a substrate (Bertsch & Müller, 2015).

## CONCLUDING REMARKS

Overflow metabolism is a mechanism for cellular resilience and most likely spans all branches of the phylogenetic tree of life. Here, we propose that overflow metabolism can also be observed in microorganisms that operate their catabolism close to thermodynamic equilibrium. The ecological niche of the microorganism could have a defining role in the actual physiology that is exploited in overflow conditions. Fundamental understanding of the drivers of product formation in CO‐consuming acetogenic microorganisms allows for process optimization independent of complex and laborious genetic engineering approaches. More specifically, we propose that the product spectrum of gas fermentation can be controlled through controlling either the specific growth rate or the biomass‐specific CO uptake rate in bioreactors.

## AUTHOR CONTRIBUTIONS

**Maximilienne T. Allaart:** Conceptualization (lead); formal analysis (equal); investigation (equal); visualization (equal); writing – original draft (lead); writing – review and editing (equal). **Martijn Diender:** Formal analysis (equal); investigation (equal); writing – original draft (equal); writing – review and editing (equal). **Diana Z. Sousa:** Funding acquisition (equal); supervision (equal); writing – review and editing (equal). **Robbert Kleerebezem:** Funding acquisition (equal); supervision (equal); writing – review and editing (equal).

## CONFLICT OF INTEREST

The authors declare no conflict of interest. This can be added.

## FUNDING INFORMATION

This study was funded by Nederlandse Organisatie voor Wetenschappelijk Onderzoek (Grant Number: P16‐10, project number 3).
